# Femoral fracture leads to moderate pulmonary organ damage in aged mice and induces immune alterations

**DOI:** 10.3389/fimmu.2026.1763857

**Published:** 2026-03-04

**Authors:** Jasmin Maria Bülow, Helen Rinderknecht, Alessa Wagner, Melanie Haffner-Luntzer, Katrin Bundkirchen, Claudia Neunaber, Borna Relja, Nils Becker

**Affiliations:** 1 Department of Trauma, Hand, Plastic and Reconstructive Surgery, Translational and Experimental Trauma Research, Ulm University Medical Center, Ulm, Germany; 2 Institute of Orthopedic Research and Biomechanics, Ulm University Medical Center, Ulm, Germany; 3 Department of Trauma Surgery, Hannover Medical School, Hannover, Germany

**Keywords:** age, cytokines, fracture, inflammation, neutrophils, Wnt

## Abstract

**Introduction:**

The relevance of age-related immunological alterations in patients experiencing fractures has drastically increased due to the global rise in life expectancy and the elevated risk of fractures among elderly individuals. The potential cross talk between long-bone fractures and the respiratory system is particularly crucial, given the high incidence of healthcare-associated pneumonia and its impact on mortality in aged patients with fractures.

**Method:**

Age-dependent differences in lung inflammation and regeneration following fracture were investigated using male C57BL/6J mice aged 17–26 weeks (young) and 64–72 weeks (aged), which underwent a unilateral femur osteotomy with external fixation (Fx) or sham surgery.

**Results:**

Fracture leads to an altered inflammatory response and expression of regeneration-associated pathways in the lung of both young and aged mice, as reflected by reduced levels of pro- and anti-inflammatory cytokines IL-6, MCP-1, and IL-10, along with increased gene expression of sclerostin, a regulator of Wnt signaling. In addition, aged mice showed increased CXCL1 levels, resulting in enhanced pulmonary neutrophil infiltration following fracture. This was associated with increased pulmonary damage, as evidenced by heightened RAGE and total protein BAL levels.

**Conclusion:**

Our data suggests that femoral fracture in the elderly impairs lung inflammatory regulation and early regeneration, which potentially increase the risk of pulmonary complications.

## 1 Introduction

The growing global influence of an aging population is highlighted by a doubled rate of elderly individuals aged 65 years and older since 1950, currently making up 10% of the world’s population (1). A further increase is expected, with rates estimated to reach up to 23% in the United States in 2050 (2). The increasing rate of elderly individuals is associated with a considerable socioeconomic burden due to their increased use of healthcare services (2). In addition to a higher prevalence of chronic diseases, the risk for injuries and bone fractures increases significantly with aging (3, 4). A recent analysis of a national registry in Germany revealed that, in 2019, around 60% of all fractures occurred in patients over 70 years of age, with proximal femoral fracture being the predominant type of fracture (5).

Risk factors for fractures in the elderly include concomitant diseases, such as osteoporosis or sarcopenia, as well as increased frailty and malnutrition (6). These factors can reduce the bone quality but also increase the risk of falls, particularly from standing height, which is one of the main causes of fractures in geriatric patients (6).

Beyond the increasing incidence of fractures, aged patients are highly endangered to develop severe post-traumatic complications (6). Several influencing factors have been described to contribute to this elevated risk, while age-related immune alterations have been increasingly recognized. Aging is associated with an altered immune response under both physiological and pathophysiological conditions (7). Research over the past decade has demonstrated impaired phagocytic capacity of innate immune cells, reduced T-cell activation, and diminished functional B-cell response in older adults (7–9). Moreover, an increase in pro-inflammatory cytokines and pro-apoptotic factors has been described in geriatric patients (7–9), contributing to a state of chronic low-grade inflammation, termed “inflammaging” (10).

These age-related immunological changes can affect both local fracture healing (11) and distant organs. It is described that the lung reacts with an increased expression of pro-inflammatory cytokines following femoral fracture and fixation (12). However, the knowledge about age-specific alteration in the pulmonal response following isolated femoral fracture is limited.

But age-associated immunological alterations may substantially contribute to a heightened risk of immune-mediated complications and infections. Especially, post-traumatic pneumonia following femoral fractures is frequently observed in elderly patients (13, 14) and has been associated with prolonged hospital stays and increased mortality rates (13, 15). The aging process leads in the lung to a change in the cellular composition of all lymphatic tissue structures, making the lungs less flexible and less responsive to infection or trauma (16, 17). In addition, decreased numbers of alveolar macrophages (AMs) and impaired T- and B-cell responses lead to weakened antiviral defenses (18–20). Elevated levels of circulating pro-inflammatory cytokines such as interleukin (IL)-1β, IL-6, IL-8, and tumor necrosis factor (TNF)-α have also been detected in the lungs of geriatric patients under physiological conditions (16, 17).

Given the known age-specific immunological differences in pulmonary physiology, the current study was designed to evaluate the impact of a standardized, isolated femoral fracture on the remote pulmonary immune responses and remote lung damage. Using both young and aged mice, we aim to assess whether age-related remote immunological alterations could contribute to a state promoting pulmonary complications following trauma. Furthermore, we assessed the role of early alteration of markers in the regenerative *Wnt* pathway. The importance of the *Wnt* pathway in pulmonary regeneration and fracture healing has been described before (21–23), while a clear age-dependent functionality is known (24).

Knowledge of risk factors and underlying immunological mechanisms for the development of complications can aid clinicians in an improved risk allocation and, furthermore, evaluate new potential therapeutic approaches.

## 2 Materials and methods

### 2.1 Animal care, husbandry, and group organization

Animals were housed in the central animal facility of the Hannover Medical School (MHH) under standardized conditions. The experiments were performed according to the ARRIVE guidelines and were approved by the ethical committee of the Lower Saxony State Office for Consumer Protection and Food Safety (LAVES; No. 33.12-42502-17/2491). Twenty-four male C57BL/6J mice (Janvier Labs, Le Genest-Saint-Isle, France) with an age of 17–26 (*n* = 12), representing a human age of 18–25 years, and 64–72 (*n* = 12) weeks, representing a human age of 60–70 years, were used for the experiments (25). Experiments were initiated earliest after one week of acclimatization after arrival in the animal facility. The mice were randomly assigned to one of the four experimental groups: sham young, Fx young, sham aged, and Fx aged (each group: *n* = 6).

### 2.2 Experimental model

For analgesia, mice received a subcutaneous injection (s.c.) of carprofen (5 mg/kg body weight, Zoetis Inc., USA) and butorphanol (1 mg/kg body weight, Zoetis Inc.) prior to surgery. Under inhalation anesthesia (Isoflurane, Baxter Deutschland GmbH, Unterschließheim, Germany), the operation site was locally anesthetized with prilocaine hydrochloride, and the femur was prepared. An external fixator (MouseExFix simple L 100%, RISystem, Davos, Switzerland) was implanted in the femoral bone of all animals. Mice in the fracture group received a standardized osteotomy at the mid-shaft of the femur, using a 0.44 mm Gigli wire saw (RISystem, Davos, Switzerland) (26). Post surgery the mice obtained metamizole (200 mg/kg body weight) in the drinking water and an s.c. injection of carprofen and butorphanol according to indication.

### 2.3 Harvesting procedures

Twenty-four hours after surgery, the mice were euthanized by intraperitoneal injection of ketamine (75 mg/kg body weight, Zoetic Inc.) and medetomidine (1 mg/kg body weight, Zoetic Inc.). Blood was obtained via cardiac exsanguination, and sacrifice was finalized by cervical dislocation. For collection of bronchoalveolar lavage (BAL) fluid, the trachea was punctured and a blunt 19-gauge syringe containing 1.1 ml phosphate-buffered saline (PBS) was inserted. The lung was flushed with the specific amount of PBS, and at least 800 µl of BAL was collected. Afterwards, BALF was centrifuged at 1,164 × g for 5 min at 4°C, and the supernatant was frozen at −80°C until further use. The trachea was closed, and the mice were perfused with prewarmed PBS through the heart by a 21-gauge blunt-tipped syringe (BD, Franklin Lakes, USA). The left lung was ligated, removed, snap-frozen in liquid nitrogen, and stored at −80°C for subsequent analysis. The right lobe of the lung was perfused with 1 ml of 4% buffered Zn-formalin solution (Thermo Fisher Scientific, Waltham, USA) through the trachea. The lobe was removed and fixed for 24h in 4% buffered formalin and transferred to 70% ethanol for storage prior to histological analyses.

### 2.4 Examination of lung damage

For hematoxylin-eosin (HE) lung sections (3 µm), from the paraffin-embedded organs were prepared. The slides were deparaffinized with Roti Histol (Carl Roth, Karlsruhe, Germany), rehydrated, and stained with Hemalum solution (Carl Roth) for 10 min at room temperature (RT). Following washing under running water for 10 min, the slides were counterstained for 3 min with eosin (Carl Roth). The slides were dehydrated by an ascending alcohol series and mounted (Mountex, Medite Medical GmbH, Burgdorf, Germany). Images were obtained using an Axio Observer Z1 microscope (40× objective, Zeiss Göttingen, Germany).

### 2.5 Measurement of total protein in BAL

Total protein in BAL was measured by bicinchoninic acid (BCA) assay (Thermo Fisher Scientific, Waltham, USA) following the manufacturer’s protocol. Briefly, a standard curve from 2,000 to 25 µg/ml was generated. Samples and standards were incubated for 30 min at 37°C with BCA working reagent (50 Reagent A:1 Reagent B). Results were quantified using the Spark M10 microplate reader (Tecan, Männedorf, Switzerland).

### 2.6 Immune histological staining of CXCL1, RAGE, neutrophil elastase, and active caspase-3

For immunohistological staining, paraffin-embedded lung tissue sections (3 µm) were used. The sections were first deparaffinized two times with Roti-Histol for 5 min and then rehydrated in a decreasing ethanol series (100%, 90%, and 70%). Epitope retrieval was performed using R-Universal epitope recovery buffer (Aptum, Kassel, Germany) with the 2100-Retriever system (Prestige Medical, Blackburn, England) at 121 °C for 20 min, following the manufacturer’s instructions. Afterwards, the slides were blocked with blocking solution (5% goat serum, 0.05% TritonX, and 0.05% Tween20 in 1 × PBS) for 20 min at RT and incubated with the following primary antibodies for one hour at RT: rabbit anti-mouse C-X-C motif chemokine (CXCL)1 (1:300; #ab269939; Abcam Inc, Toronto, ON, Canada), rabbit anti-mouse advanced glycation end-products (RAGE) (1:100, #ab3611, Abcam Inc, Toronto, ON, Canada), rabbit anti-mouse neutrophil elastase (NE) (1:200; #bs-6982R; Bioss, USA), and rabbit anti-mouse active caspase-3 (Asp175) (1:300; #9661; Cell Signaling Technology, USA). Antibodies were diluted in Antibody Dilution Buffer (DakoCytomation) as recommended by the manufacturer. Afterwards, endogenous peroxidase activity was inhibited by 3% hydrogen peroxide for 15 min at RT.

Goat anti-rabbit IgG-HRP (#414341F; Histofine Simple Stain Mouse MAX PO (R), Nichirei Biosciences Inc., USA) was used as a secondary antibody and incubated for 60 min at RT. For signal detection, 3-amino-9-ethylcarbazol (AEC, DCS Innovative Diagnostik-Systeme, Hamburg, Germany) was applied, and the sections were counterstained with hematoxylin (Carl Roth) and mounted (Mountex, Medite Medical GmbH Burgdorf, Germany). Images were obtained using an Axio Observer Z1 microscope (40× objective, Zeiss Göttingen, Germany). For evaluation, the ImageJ software was used. Neutrophil elastase and caspase-3 positively stained cells were counted in 25 high-power fields per animal in the 40× magnification. CXCL1 and RAGE staining were evaluated by determination of the mean intensity values.

### 2.7 Gene expression analysis

RNA from lung tissue was isolated by mechanical homogenization using the Precellys 24 Homogenizer (Bertin Technologies, Montigny-le-Bretonneux, France) and the RNeasy Mini Kit, according to the manufacturer’s protocol (Qiagen, Hilden, Germany). RNA concentration and quality were measured by the Tecan Spark M10 Microplate Reader (Tecan, Männedorf, Switzerland). By using the iScript™ cDNA synthesis kit (BioRad, Hercules, USA), RNA was transcribed into cDNA. The synthesized cDNA was used for quantitative real-time PCR (qRT-PCR) analysis. SYBR Green qPCR Master Mix (BioRad) was used for qRT-PCR in a total reaction volume of 25 μl and run in a CFX96 Touch Real-Time PCR Detection System (BioRad). Gene expression of *Sost* (#qMmuCED0045167; BioRad) and *Wnt3a* (#qMmuCID00055162; BioRad) was analyzed using the ΔΔCT method. Values were normalized to the housekeeping gene *Gapdh* (qMmuCED0027467; BioRad) and to the respective controls.

### 2.8 Protein expression levels via ELISA

Lung tissue was homogenized in lysis buffer (#FNN0021, Invitrogen, Waltham, USA) at 4°C and centrifuged at 20,000 × g for 30 min at 4°C. Supernatants were collected and stored at −80°C until further use. Tissue cytokine concentrations of IL-10, IL-6, and monocyte chemoattractant protein (MCP)-1 were analyzed using mouse-specific ELISA kits (R&D Systems, Minneapolis, MN, USA) following the manufacturer’s protocol. Results were quantified using the Spark M10 microplate reader (Tecan, Männedorf, Switzerland).

### 2.9 Statistics

The results were displayed as a bar chart showing mean ± standard error of the mean. Data were tested for normal distribution using the Shapiro-Wilk normality test. As the data was not normally distributed a Mann-Whitney U test without adjustments was performed. Values of *p* < 0.05 were considered as statistically significant. *P*-values for trends are exact values from the test. Statistical analysis was performed using Graph Pad Prism 10.0 software (GraphPad Software, La Jolla, CA, USA).

## 3 Results

### 3.1 Fracture leads to a moderate remote lung damage in aged mice

**Figure 1:**
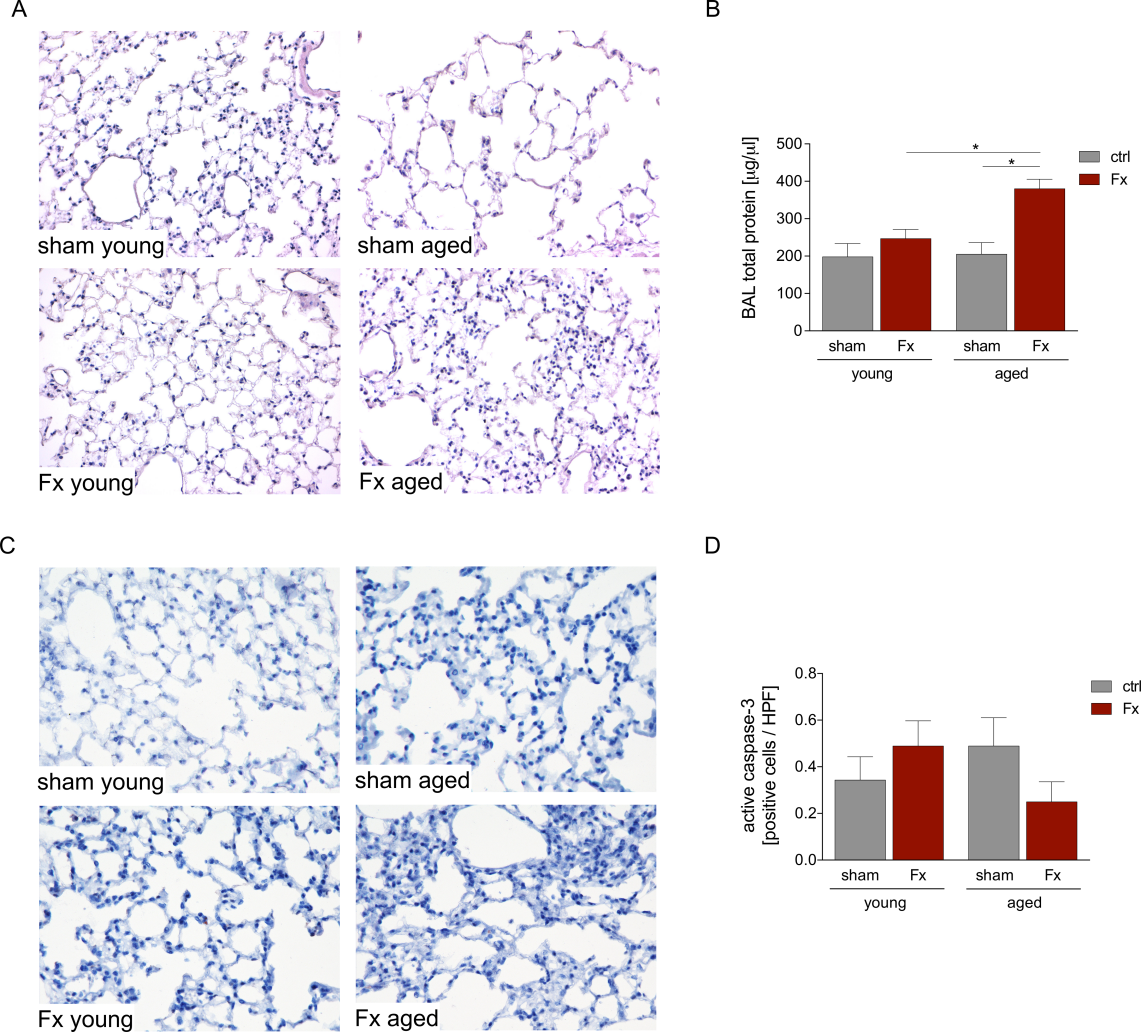
Lung damage in young and aged mice after femur fracture. Young (17–26 weeks) and aged (64–72 weeks) mice received a unilateral femur fracture or sham surgery. Twenty-four hours post-surgery, mice were euthanized, and sampling was performed. **(A)** Representative images of hematoxylin-eosin (HE) stained lung sections in a 40× magnification. **(B)** Concentration of total protein in bronchoalveolar lavage (BAL) fluid. **(C)** Representative images in a 40× magnification and **(D)** quantitative analysis of active caspase-3 expression in the lung. Data are presented as mean ± standard error of the mean; n = 6 per group. * = p < 0.05.

In this experiment, young (17–26 weeks) and aged (64–72 weeks) mice received a unilateral femur fracture or sham surgery. Lung damage after fracture (Fx) was evaluated by HE staining (**Figure 1A**) and the total protein concentration in the BAL fluid (**Figure 1B**).

In young mice, femur fracture did not affect the total protein concentration in the BAL fluid. In contrast, aged mice showed a significantly increased total protein concentration after fracture compared to the sham-aged group and the young fracture group (**Figure 1B**).

Quantification of apoptosis via counting of active caspase-3-positive cells indicated comparable cell numbers between all groups (**Figures 1C, D**) findings that were reflected by the histopathological (HE) overall tissue damage assessment (**Figure 1A**).

### 3.2 Increased neutrophil infiltration after fracture in aged mice

**Figure 2:**
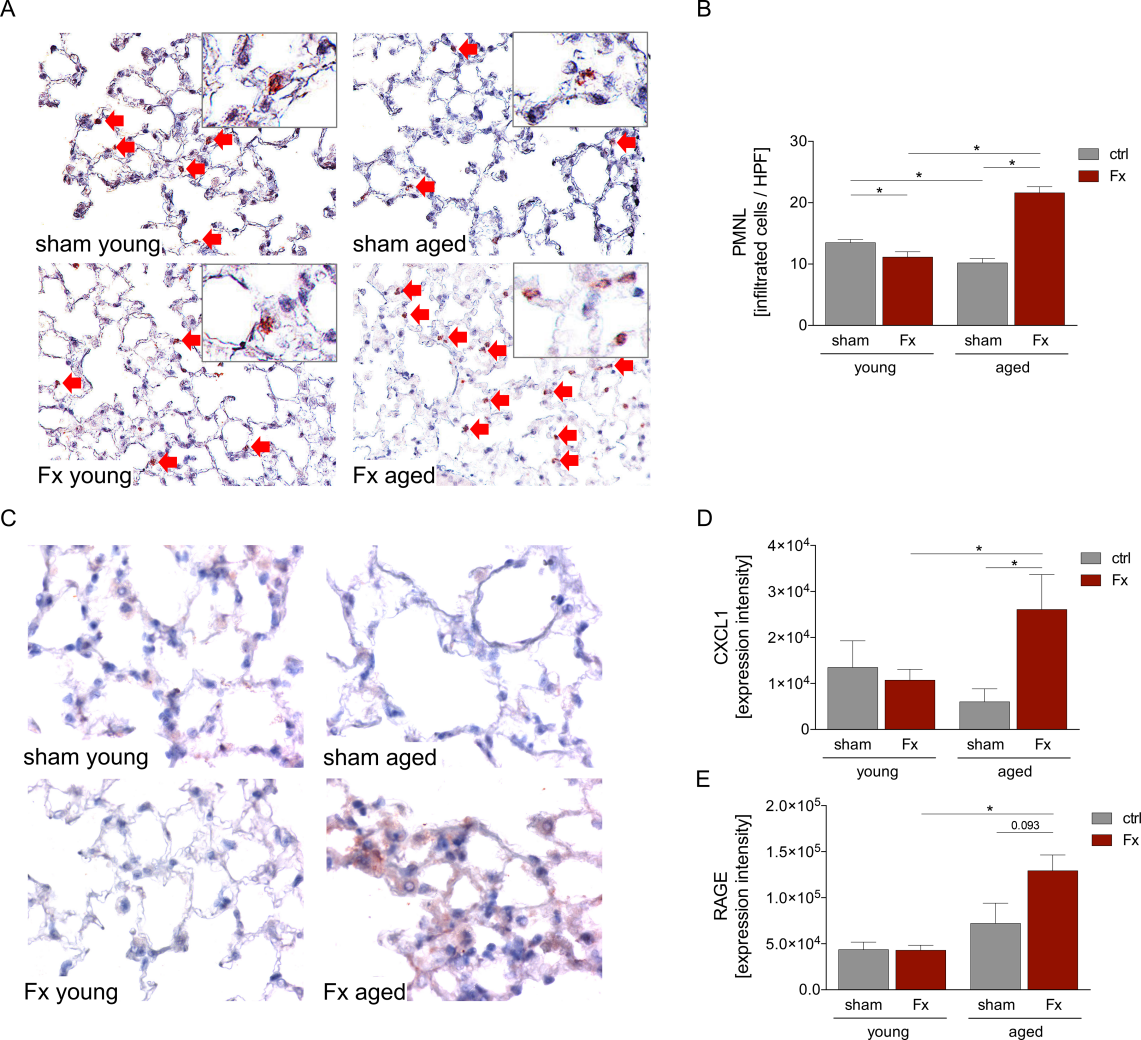
Aging affects pulmonary neutrophil infiltration and cytokine levels after femoral fracture. Young (17–26 weeks) and aged (64–72 weeks) mice received a unilateral femur fracture or sham surgery. Twenty-four hours post-surgery, mice were euthanized, and sampling was performed. **(A)** Representative immune histological staining of neutrophil-elastase (NE) as a marker of PMNL in lung sections in a 40× magnification. **(B)** Quantification of NE-positively stained cells per high-power-field (HPF) in the lung. **(C)** Representative immune histological staining of CXCL1 in lung sections in a 40× magnification and **(D)** quantitative analysis of the expression intensity. **(E)** Quantitative analysis of receptor for advanced glycation end products (RAGE) in lung. Data are presented as mean ± standard error of the mean; n = 6 per group. * = p < 0.05.

To further investigate lung inflammation in young and aged mice post-fracture, the infiltration of polymorphonuclear leukocytes (PMNL), specifically neutrophils, was determined (**Figures 2A, B**). For this purpose, neutrophil elastase (NE) staining was performed in the lungs.

Quantification of immunohistochemical staining revealed a decreased infiltration of PMNL in lung tissue after fracture in young mice compared to the corresponding sham group. In contrast, a significantly increased PMNL infiltration was observed in aged mice after fracture. Comparison of the PMNL infiltration between young and aged mice showed a significantly reduced PMNL number in the sham-aged group but an increased number of PMNL after fracture (**Figures 2A, B**).

Infiltration of PMNL to the site of inflammation is promoted by various cytokines such as CXCL1. Immunohistochemical evaluation of CXCL1 expression intensity showed a slight but not statistically significant reduction in the young Fx group compared to the sham group. Whereas the expression intensity was significantly increased in aged mice post-fracture compared to the aged sham group. Comparable to the PMNL infiltration, CXCL1 expression was slightly decreased in the sham-aged group compared to the sham young group, whereas after fracture a significantly increased expression intensity was observed in aged mice compared to young mice (**Figures 2C, D**).

To further investigate the pulmonary inflammatory response after femoral fracture, the expression intensity of RAGE was quantified. Similar results as for CXCL1 expression were obtained, despite a trend to an increased expression in aged sham compared to young sham animals. In the young group no difference between sham and fractured mice was observed. In the aged group, a trend to an increased RAGE expression intensity was shown post-fracture (*p* > 0.093, **Figure 2E**). Furthermore, the comparison to the young fracture group displayed a significant increased RAGE expression intensity in the aged fracture group (**Figure 2E**).

### 3.3 Fracture influences lung inflammation in young and aged mice

**Figure 3:**
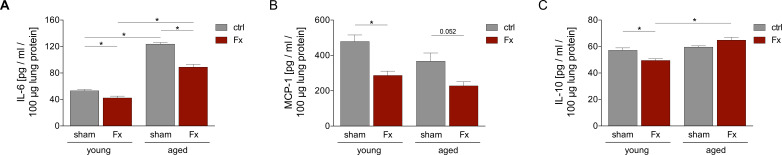
Fracture modulates the pulmonary inflammation. Young (17–26 weeks) and aged (64–72 weeks) mice received a unilateral femur fracture or sham surgery. Twenty-four hours post-surgery, mice were euthanized, and sampling was performed. Protein level of **(A)** IL-6, **(B)** MCP-1, and **(C)** IL-10 in lung tissue homogenates. Data are presented as mean ± standard error of the mean; n = 6 per group. * = p < 0.05.

To investigate the impact of a femoral fracture on pulmonary inflammation, protein levels of pro- and anti-inflammatory cytokines in the lung were evaluated (**Figure 3**).

The protein level of IL-6, a key player in triggering inflammation after trauma, was significantly reduced after fracture in young and aged mice compared to the corresponding sham groups. Yet, IL-6 levels were two- to threefold increased in both aged groups (sham and Fx) compared to their young counterparts (**Figure 3A**).

Fracture reduced the protein level of MCP-1, a regulator of monocyte/macrophage infiltration, in young mice significantly, and in aged mice with a strong trend (aged *p* > 0.052). However, no significant differences between young and aged fractured mice were observed (**Figure 3B**).

The anti-inflammatory cytokine IL-10 was significantly reduced after fracture in the lung tissue of young mice compared to young sham mice. In aged mice, fracture did not influence the pulmonary IL-10 protein level, though there was a trend to an increase. However, aged mice showed a significantly increased IL-10 level post-fracture compared to the young fractured group (**Figure 3C**).

### 3.4 Fracture modulates pulmonary Wnt signaling

The observed age-dependent inflammatory responses to traumatic injury suggest a further involvement of early regulators of lung regeneration. As a significant factor for recovery following lung injury, the Wnt pathway has been described as an important pathway (21). Furthermore, age-dependent functional alterations of the Wnt pathway have been described (24).

**Figure 4:**
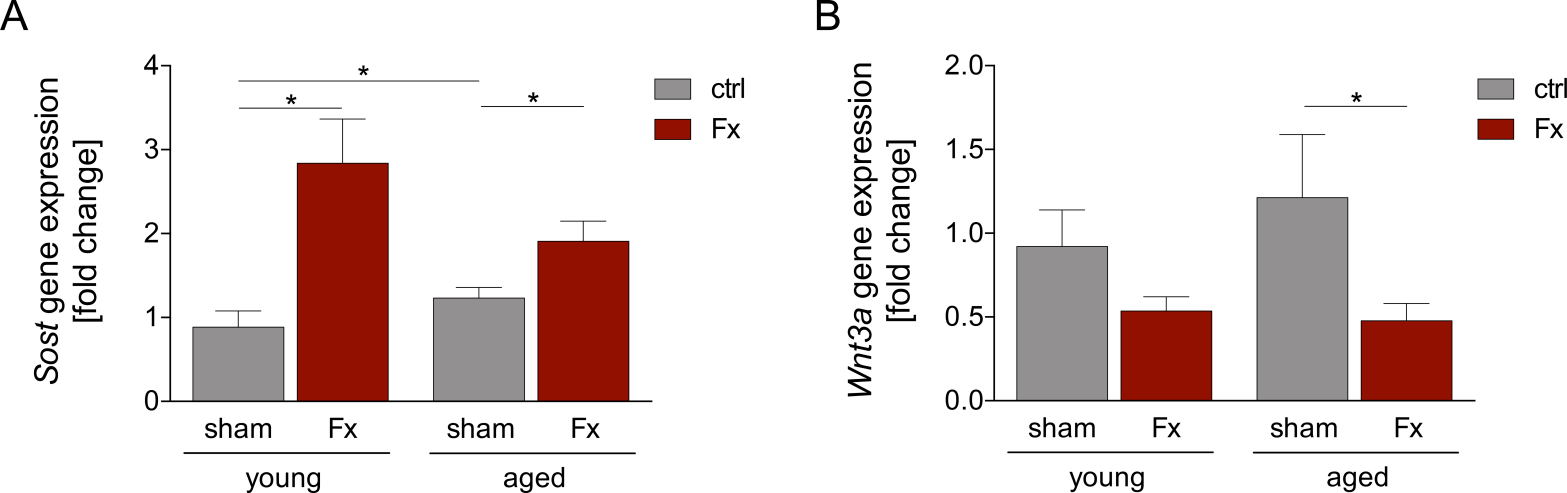
Wnt signaling in the lung is modulated after femoral fracture. Young (17–26 weeks) and aged (64–72 weeks) mice received a unilateral femur fracture or sham surgery. Twenty-four hours post-surgery, mice were euthanized, and sampling was performed. Gene expression analysis of **(A)** Sost and **(B)** Wnt3a in lung tissue homogenate. Data are presented as mean ± standard error of the mean; n = 6 per group. * = p < 0.05.

To address our hypothesis that isolated femoral fractures induce the Wnt pathway predominantly in young mice, gene expression analysis of the Wnt pathway was conducted (**Figure 4**).

Gene expression of *Sost*, an antagonist of the Wnt signaling, was significantly upregulated after fracture in both young and aged groups compared to the corresponding sham animals. Interestingly, the sham-aged group showed a significantly increased expression of *Sost* compared to the sham young group (**Figure 4A**). A trend to a decreased expression of *Sost* in aged, fractured mice was observed compared to the young fractured mice.

Analysis of the *Wnt3a* gene expression, a ligand of the canonical Wnt signaling pathway, showed reduced expression after fracture in both age groups. However, this reduction was only significant in aged animals (**Figure 4B**). There were no significant differences between young and aged mice for both treatment groups.

## 4 Discussion

In this study, we demonstrated that an isolated femur fracture leads to altered inflammation in the lungs of both young and aged mice. Aged mice showed increased infiltration of neutrophils following fracture. Furthermore, there are hints towards an impaired regenerative potential, as we found an altered regulation of the Wnt signaling. Overall, our data suggests that the aging process contributes to an altered pulmonary inflammatory and regenerative response after femoral fracture, which might increase the risk of infections and complications.

Fractures are associated with the disruption of bone structure, blood vessels, and surrounding tissue, which promotes both local and systemic inflammation due to the release of damage-associated molecular patterns (DAMPs) and various cytokines into circulation (27–29). Geriatric patients are at risk for delayed and impaired fracture healing, resulting from a delayed immune response, impaired vascularization, and altered bone remodeling (30, 31). Furthermore, altered post-traumatic inflammation, as well as a generally present low-grade inflammation in the elderly individuals, may affect distant organs beyond the site of injury (32, 33). Yet, little is known about the distinct remote effects of an isolated fracture on other organ systems.

The potential impact on a patient’s clinical course and post-traumatic quality of life could be tremendous, given the high rates of complications in elderly patients with fractures and the associated hospitalization and mortality (13, 34). Especially regarding the reduced physiological resources in elderly individuals (35), even minor deterioration of organ functions can lead to significant and life-threatening complications. Considering the aging population (2) and the high rate of older adults presenting with fractures (5), several patients could be affected by age-specific immune alterations, potentially promoting post-traumatic complications.

Therefore, in this study, we assessed the effect of femoral fractures on key pulmonary immune reactions using an experimental mouse model that differentiates between young and aged mice. We focused on the influence of fracture on pulmonary response, as pneumonia is one of the most observed complications after fracture (13, 34), while patients with femoral fractures are particularly at risk, given their often impaired and delayed mobilization (36).

However, here, we were not able to detect significant histopathological signs of lung damage following isolated femoral fracture in young mice. In aged mice, however, we observed an increased amount of protein in the BAL fluid after fracture, which is an indicator for pulmonary tissue damage (37). In addition, HE staining shows an increased number of cells in the lungs of aged, fractured mice, as well as a thickening of the alveolar septum. Nevertheless, no differences in apoptosis, measured by caspase-3 positive cells, were found. These findings align with previous studies showing increased lung damage after trauma consisting of femoral fracture and hemorrhagic shock or ischemia-reperfusion injury of the lung in aged mice (38, 39). The observed remote lung damage, seen exclusively in aged mice, could contribute significantly to the elevated risk of pulmonary complications in aged patients with fractures (40). Fracture-induced additional lung damage could especially be detrimental for patients with pre-existing pulmonary diseases, such as chronic obstructive pulmonary disease (COPD). A study by Cha et al. reported COPD in 34% of patients over 65 years with hip fractures, with significantly increased short- and long-term mortality (41). To assess potential contributors to the observed organ injury, we focused on the pulmonary infiltration of PMNL into the lungs. An increased infiltration of PMNL has repeatedly been correlated with an increased pulmonary damage (42), due to PMNL degranulation, generation of reactive oxygen species (ROS), or the release of neutrophil extracellular traps (43).

Interestingly, while Zhou et al. demonstrated increased infiltration of PMNL into the lung tissue after femoral fracture and hemorrhagic shock in young and aged mice (38), we could only demonstrate an increased number of neutrophils following femur fracture in aged mice. In contrast, we observed a decline in PMNL infiltration after fracture in young mice. This could be explained by the fact that Zhou et al. induced an additional hemorrhagic shock (HS), which causes increased immune response and damage in various organs (33, 38, 44). In addition, faster clearance of neutrophils might also be the underlying cause of reduced PMNL after fracture in young mice. However, to confirm this, further time points and PMNL-subset analysis in lung tissue and BAL fluid are needed (45).

A critical chemokine involved in the recruitment and migration of PMNL to the site of injury is CXCL1 (46–50). The importance of CXCL1 in the lung was demonstrated by Vollrath et al. (51), which showed an increase in CXCL1 following acute lung injury. Here, we showed elevated CXCL1 levels after fracture in aged mice, correlating with the observed increased infiltration of PMNL into the lung tissue. Furthermore, we assessed the expression of RAGE, which is highly expressed in the lung tissue and plays an important role in lung homeostasis and different pulmonary diseases (52). RAGE is also a major mediator of the inflammatory reaction in the lung (52, 53). In aged mice, fracture led to increased RAGE expression in the lungs, which also correlated with PMNL infiltration and CXCL1 levels. This suggests an increased inflammatory response in aged mice following fracture.

Although aged mice exhibited increased levels of CXCL1 and RAGE, the concentrations of other pro-inflammatory markers such as IL-6 or MCP-1 were decreased in both young and aged mice following fracture. As PMNL levels were similarly decreased in younger mice, it has been shown, that PMNL in aged mice show a decreased ability for cytokine signaling (54). These age-specific cellular changes could have contributed to the unchanged level of IL-6 despite the significant PMNL infiltration. Also, in this study we did not assess phenotypic subsets of the infiltrating PMNL to the lungs, yet it has been described that PMNL of elderly individuals have higher rates of CD16^bright^/CD62L^dim^ PMNL. These cells are associated with a reduced ability for phagocytosis and ROS production (55). Potential phenotypical age-dependent alterations of the infiltrating PMNL could therefore further contribute to increased lung damage with concomitant increased risk for pulmonary infection, given the decreased antimicrobial functions of CD16^bright^/CD62L^dim^ PMNL. Furthermore, stimulus-dependent changes in the rates of PMNL subsets have been described in tissue directly affected by trauma compared to tissue that has not been damaged (45), which could also have contributed to the observed results. However, no functional assessment of PMNL has been performed in this study. Further investigation of the PMNL subsets is necessary to clearly identify the role of the increased pulmonal PMNL infiltration.

Interestingly, aged mice showed a generally increased level of IL-6 in both groups (sham and Fx) compared to young mice, supporting the low-grade inflammation status in elderly individuals. However, compared to our study, other studies demonstrate an increase in IL-6 level after trauma (38, 56, 57). The combination with other traumata, such as hemorrhagic shock or thoracic trauma, seems to be crucial for an increased pulmonary IL-6 level (38, 56, 57). Fracture-induced changes in MCP-1 were also observed, with reduced concentrations in young mice and a downward trend in aged mice. MCP-1 plays a crucial role in the recruitment and activation of monocytes after trauma but also in different pulmonary diseases (58, 59). Monocytes are essential during the resolution of inflammation and can promote pulmonary regeneration following lung inflammation (60). Thus, the combination of fracture-induced decreased MCP-1 levels and age-specific remote lung injury may additionally impair restoration of the pulmonary function for a prolonged time.

Moreover, young mice showed a decrease in IL-10 levels after fracture, whereas aged mice showed a relative increase. IL-10 is an important cytokine in the suppression of inflammation in the lung (61); thus, altered pulmonary IL-10 levels after fracture may indicate an impaired anti-inflammatory response.

Regeneration of lung tissue after trauma is an important process for restoring lung functions. A key pathway involved in this process is the Wnt signaling (62, 63). Wnt signaling has been described to be reduced in the lungs of older individuals (24), while a high induction of the pulmonary Wnt pathway was associated with elevated lung injury (21). Given this background, we hypothesized that a remote injury would interfere with the age-dependent activation of *Wnt* signaling and the occurrence of acute lung injury. Therefore, we analyzed the gene expression of Wnt regulators. In both young and old animals, fracture led to an increased expression of sclerostin, a Wnt pathway antagonist (64). Furthermore, expression of the Wnt activator Wnt3a was reduced. These results suggest altered early Wnt signaling following fracture, which may impair pulmonary regeneration. However, further investigations of the Wnt signaling at different time points are needed to fully understand the effect of altered Wnt signaling on lung recovery.

Our results indicate that fracture-associated remote effects impair pulmonary regeneration in an age-dependent manner. These findings may have important clinical implications, especially given the rise of aged patients presenting with femoral fractures (2). Identifying the underlying mechanisms driving these effects is essential for developing targeted strategies to prevent complications.

Furthermore, clinical relevance could also arise through the combined impact of the age-dependent immune reaction with treatment-induced pulmonary effects. The utilization of bone cement in osteoporotic bone is common during operative procedures. Adverse effects of bone cementation (65) and age-dependent pulmonal effects could combind lead to an increased risk for pulmonal complications.

Despite these insights, some important limitations of this study have to be stated that could limit the clinical translation of our findings. First, we focused solely on local pulmonary inflammation without evaluating systemic inflammation or serum markers of lung injury. Assessing these parameters could provide a broader understanding of inflammatory dynamics.

Second, the experimental study design was restricted to a single early post-trauma time point (24h). As inflammatory and regenerative responses are dynamic processes, additional time points (both earlier and later) are essential to fully characterize the lung response and regeneration capacity after fracture. Further studies addressing the molecular regulation of inflammation and regeneration are needed to fully understand the effect of fracture on the lung. Third, the study included only male mice, based on data indicating a higher risk of pneumonia in male fracture patients (66). Given known sex-specific differences in immune response to trauma (67), future studies should include female mice to determine sex-specific differences. Animal models, although valuable, do not directly replicate human responses; thus, extrapolation of these findings to clinical settings requires careful consideration and validation through clinical studies. These studies should also include the possible influence of pre-existing medical conditions and medication, which are frequently observed in aged patients. Future investigations addressing these limitations are critical for fully elucidating the complexity of inflammatory and regenerative processes following trauma.

In conclusion, this study demonstrates for the first time that an isolated femoral fracture induces significant inflammatory and regenerative changes in the lungs, particularly in aged mice. These age-dependent alterations suggest that older patients may be at increased risk for pulmonary complications following fracture. Further studies are needed to elucidate our understanding of this process, particularly underlying molecular mechanisms, to optimize the clinical management of geriatric patients post-fracture.

## Data Availability

The original contributions presented in the study are included in the article/supplementary material. Further inquiries can be directed to the corresponding author.
